# Screening and Identification of Key Biomarkers for Bladder Cancer: A Study Based on TCGA and GEO Data

**DOI:** 10.1155/2020/8283401

**Published:** 2020-01-23

**Authors:** Yingkun Xu, Guangzhen Wu, Jianyi Li, Jiatong Li, Ningke Ruan, Liye Ma, Xiaoyang Han, Yanjun Wei, Liang Li, Hongge Zhang, Yougen Chen, Qinghua Xia

**Affiliations:** ^1^Department of Urology, Shandong Provincial Hospital Affiliated to Shandong University, Jinan, China; ^2^Department of Urology, The First Affiliated Hospital of Dalian Medical University, Dalian, China; ^3^Department of Nephrology, Shandong Provincial Hospital, Shandong First Medical University, Shandong Academy of Medical Sciences, Jinan, China; ^4^The Nursing College of Zhengzhou University, Zhengzhou, China; ^5^Department of Liver Transplantation and Hepatobiliary Surgery, Shandong Provincial Hospital Affiliated to Shandong University, Jinan, China; ^6^Department of Oncology, Shandong Provincial Hospital Affiliated to Shandong University, Jinan, China; ^7^Shangdong Academy of Medical Sciences, Jinan University, Jinan, China; ^8^Department of Urology, Tengzhou Hospital of Traditional Chinese Medicine, Tengzhou, China

## Abstract

Bladder cancer (BLCA) is a common malignant cancer, and it is the most common genitourinary cancer in the world. The recurrence rate is the highest of all cancers, and the treatment of BLCA has only slightly improved over the past 30 years. Genetic and environmental factors play an important role in the development and progression of BLCA. However, the mechanism of cancer development remains to be proven. Therefore, the identification of potential oncogenes is urgent for developing new therapeutic directions and designing novel biomarkers for the diagnosis and prognosis of BLCA. Based on this need, we screened overlapping differentially expressed genes (DEG) from the GSE7476, GSE13507, and TCGA BLCA datasets. To identify the central genes from these DEGs, we performed a protein-protein interaction network analysis. To investigate the role of DEGs and the underlying mechanisms in BLCA, we performed Gene Ontology (GO) and Kyoto Gene and Genomic Encyclopedia (KEGG) analysis; we identified the hub genes via different evaluation methods in cytoHubba and then selected the target genes by performing survival analysis. Finally, the relationship between these target genes and tumour immunity was analysed to explore the roles of these genes. In summary, our current studies indicate that both cell division cycle 20 (CDC20) and abnormal spindle microtubule assembly (ASPM) genes are potential prognostic biomarkers for BLCA. It may also be a potential immunotherapeutic target with future clinical significance.

## 1. Introduction

Bladder cancer (BLCA) is a serious health problem worldwide, and it is the second most common malignant tumour of all genitourinary tract tumours [1]. Risk factors for BLCA are known to include tobacco, schistosomiasis, eating habits, and lifestyle. In 2012, approximately 430,000 new cases of BLCA were diagnosed [2]. In China, the rate of BLCA occurrence increased rapidly during the five-year period from 2003 to 2008, and the growth rate in women was higher than that in men [3]. Despite surgery, dissection, and various adjuvant treatments for BLCA, the five-year survival rate is still low, and the risk of recurrence is high. According to reports, 30–70% of tumours reoccur [4] and 30% of tumours develop into muscle-invasive diseases [5]. Therefore, there is an urgent need to discover new and reliable BLCA biomarkers.

In recent years, immunotherapy has become the focus of cancer treatment strategies. Immunotherapy-related drugs have been approved for marketing and became available recently for asymptomatic or very mildly symptomatic prostate cancer [6], unresectable or metastatic melanoma [7], advanced melanoma, and acute lymphocytic leukaemia [8]. The immune response to cystatin has been confirmed very early [9]. Intravesical instillation of BCG can kill bladder tumour cells by inducing the infiltration of cytotoxic T lymphocytes (CTL) in NMIBC patients [10]. Recently, it was reported that anti-PD-1/PD-L1 antibodies affect the growth of tumour cells by acting on T cells [11]. At present, there is an increasing number of studies on the role of immune checkpoints and immune cells in influencing tumour development. Therefore, it is necessary to find new possible prognostic and immunotherapeutic biomarkers for BLCA.

To identify potential biomarkers for BLCA, we performed a series of analyses based on high-throughput sequencing data obtained from three data sets, GSE7476, GSE13507, and TCGA BLCA. We first identified the DEGs that are common among the three databases, as the combination of multiple databases can provide more credible results. Then, we used the Metascape website and the online tool from the DAVID website to analyse GO and KEGG terms, explore the main pathway of DEG enrichment, and explore the research progress on the pathway in bladder cancer. The protein interaction network between DEGs was constructed by using the online tool from the STRING website and illustrated with Cytoscape software. Then, we used the cytoHubba plugin for Cytoscape to search for the hub gene. Here, we used four different models, DEGREE, MCC, DMNC, and MNC, to screen out the most significant hub genes. We then used the Gene Expression Profiling Interactive Analysis (GEPIA) and Human Protein Atlas online tools to explore genes in the hub gene network that are associated with bladder cancer prognosis. Finally, we used the UALCAN, cBioPortal, STRING, Cytoscape, and TIMER tools to explore this single gene and its main biological role. We demonstrate that CDC20 and ASPM are possible biomarkers for BLCA. After further exploration, we were pleasantly surprised to find that both CDC20 and ASPM are associated with the prognosis and immunotherapy response of patients with BLCA. In vitro, we interfered with the expression of ASPM and CDC20 and then used the cell counting kit-8 experiment and clone formation experiment to detect the effect on the proliferation of bladder cancer T24 cell line. In summary, our study provides new potential prognostic markers and potential immunotherapeutic targets for BLCA.

## 2. Materials and Methods

### 2.1. Microarray Data

GEO (https://www.ncbi.nlm.nih.gov/geo/) is a database containing high-throughput gene expression data, chips, and microarrays [12]. We downloaded a gene expression dataset (GSE7476) from GEO (Affymetrix GPL3111 platform). This gene expression dataset was translated into commonly used gene symbols by using annotation information from the platform. The GSE7476 dataset contained 9 BLCA tissue samples and 3 noncancer samples.

### 2.2. Data Processing

GEO2R (http://www.ncbi.nlm.nih.gov/geo/geo2r/) is an analysis tool that comes with the GEO database and is used to compare two sets of data; it can be used to analyse any GEO series. Since the GSE13507 dataset does not contain CELL subfiles that can be analysed by R language, we chose to use GEO2R to screen differentially expressed mRNA between normal tissue samples and cancer tissue samples in the GSE13507 dataset [13]. *P* < 0.05 and logFC > 1 or < –1 were set as the cut-off criteria. The GSE13507 dataset contained 188 BLCA tissue samples and 68 noncancer samples.

### 2.3. The DEGs in BLCA from TCGA

TCGA is a vast repository of high-throughput data on DNA, RNA, and proteins in a variety of human cancers, which enables a complete analysis of the expression of these components in various cancer types. In the current study, we obtained mRNA expression profiles from BLCA and adjacent normal tissues from GEPIA (TCGA Data Online Analysis Tool) (http://gepia2.cancer-pku.cn/#index) [14]. The TCGA dataset contained 404 BLCA tissue samples and 19 noncancer samples.

### 2.4. Functional and Pathway Enrichment Analyses

First, we performed Gene Ontology (GO) and Kyoto Encyclopedia of Genes and Genomes (KEGG) analysis on DEGs by using the Metascape software (http://metascape.org/gp/index.html#/main/step1) [15]. Metascape is an online analysis tool with integrated discovery and annotation capabilities. To ensure the credibility of the results, we also analysed the data with online tools from the DAVID website and visualized the results via the R language. The DAVID website (https://david.ncifcrf.gov/home.jsp) is a bioinformatics data resource composed of a comprehensive biological knowledge base and analytical tools [16]. A *P* value < 0.05 was set as the cut-off criterion.

### 2.5. Protein-Protein Interaction (PPI) Network Construction and Module Analysis

STRING (https://string-db.org/) can draw PPI networks after importing common DEGs into search tools to retrieve interacting genes [17]. First, we drew the PPI network diagram of DEGs by using the STRING website. Cytoscape, a free visualization software, was applied to visualize PPI networks and find hub genes [18]. Then, the hub genes were identified by four methods: DEGREE, MCC, DMNC, and MNC in cytoHubba [19].

### 2.6. Association of Hub Genes Expression with the Survival of Patients with BLCA

The GEPIA website can provide fast and customizable functions based on TCGA data. We first analysed the expression of the target gene using the GEPIA website and then analysed the prognosis of the target gene using the Human Protein Atlas website (https://www.proteinatlas.org/) [20]. The results from the two websites were used to find the target gene. *P* < 0.05 was considered statistically significant.

### 2.7. Analysis of Target Genes

First, through the TIMER website, the expression of the target gene in various tumours was found. Then, the UALCAN website (http://ualcan.path.uab.edu/analysis.html) was used to determine which factors are related to the expression of the target gene in BLCA. Then, the STRING website was queried for the ten genes with the highest correlation with the target gene. In addition, Cytoscape software was used to map the protein interactions and the enrichment pathways. Finally, these data were imported into the cBioPortal website (https://www.cbioportal.org/) to query the variation in BLCA [21]. The cBioPortal website was used to analyse coexpression of the target gene and other genes in the enrichment pathway.

### 2.8. Correlation between mRNA Expression and Immune Cell Infiltration and Immune Checkpoints

TIMER (https://cistrome.shinyapps.io/timer/) is a website dedicated to analysing tumour immune relatedness. We used TIMER to analyse the mRNA expression data for CDC20 and ASPM in TCGA BLCA tumour samples and its correlation with tumour infiltration of 6 immune cell types (B cells, CD4+ T cells, CD8+ T cells, neutrophils, macrophages, and dendritic cells) and 5 immunological checkpoints (PDCD1, CD274, PDCD1LG2, TOX, and CTLA4) [22].

### 2.9. Cell Culture and Reagents

The human bladder cancer cell line T24 was purchased from the Chinese Academy of Sciences cell bank. Cells were incubated at 37°C with 5% CO_2_ in RPMI 1640 medium (Gibco BRL, Rockville, MD) supplemented with 10% heated-inactivated fetal bovine serum (FBS, Biological Industries, Kibbutz Beit HaEmek, Israel) and 1% penicillin-streptomycin (Macgene, Beijing, People's Republic of China).

### 2.10. Proliferation Analysis

Bladder cancer cell line (T24) was transfected with siASPM, siCDC20, or siControl in 24-well plates. After 24 h, the cells were seeded into 96-well plates. Cell viability was then measured using Cell Counting Kit-8 (CCK8) every 24 h. For the clone formation assay, cells were plated in a six-well plate (500 cells per well). After 2 weeks, the cells were fixed with 4% paraformaldehyde for 2 h, stained with 1% crystal violet. All assays were conducted more than three times.

### 2.11. Statistical Analysis

The data are expressed as the mean ± S.E.M. Statistical analysis was performed using Prism software (GraphPad, CA, USA). Statistical significance of differences between and among groups was assessed using the *t*-test. Significant differences are indicated as follows: ^*∗*^*P* < 0.05; ^*∗∗*^*P* < 0.01; ^*∗∗∗*^*P* < 0.001.

## 3. Results

### 3.1. Identification of DEGs in BLCA

**Figure 1:**
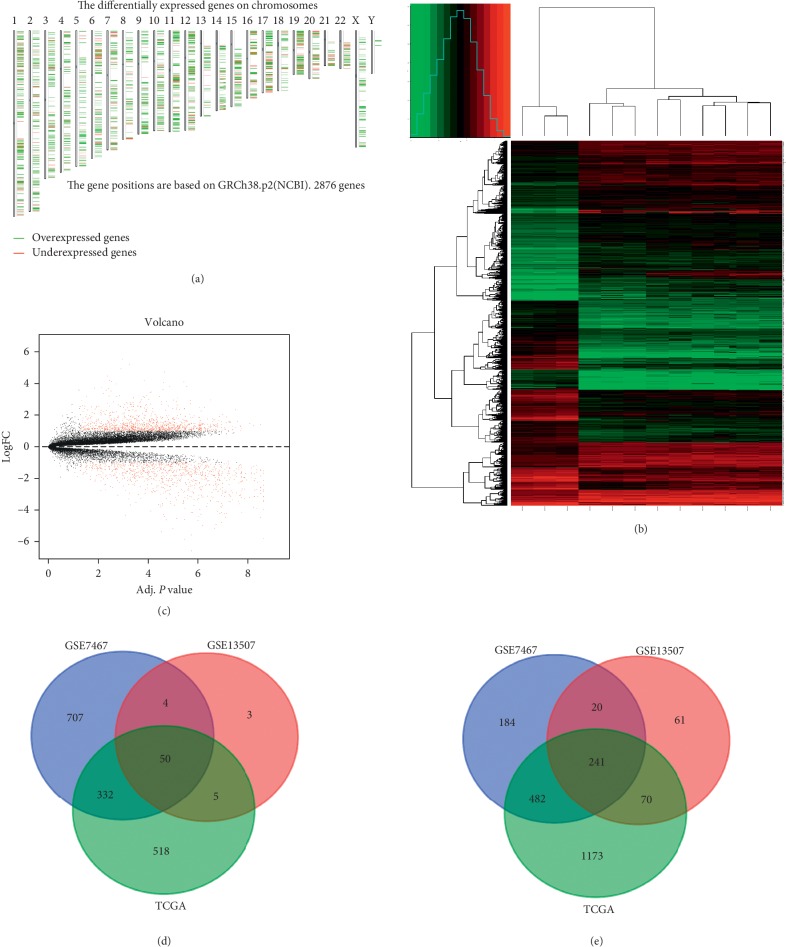
Identification of DEGs shared between the three databases. (a) The differentially expressed genes on chromosomes. (b) The heat map of GSE7467. (c) The volcano map of GSE7467. (d) A Venn diagram used to identify 50 promising upregulated target genes in BLCA. (e) A Venn diagram used to identify 241 promising downregulated target genes in BLCA.

We found the differentially expressed genes on chromosomes of BLCA cells through the GEPIA website (Figure 1(a)). R studio was used to investigate the DEGs via mining of the GEO (GSE7476) database (https://www.ncbi.nlm.nih.gov/geo/). We analysed the DEGs in the database and showed them in a heat map (Figure 1(b)) and a volcano map (Figure 1(c)). In addition, we used GEO2R to compare cancer and normal tissues in the GEO (GSE13507) and identify genes that were differentially expressed in this dataset. Then, we explored BLAC DEGs via the GEPIA website based on the TCGA database. The data were filtered by logFC > 1 or < –1 and *P* < 0.05. The overlapping DEGs among the 3 datasets were identified, and 50 upregulated genes (Figure 1(d)) and 241 downregulated genes (Figure 1(e)) were selected and presented using a Venn diagram. Fifty upregulated and 241 downregulated DEGs are listed in Table 1.

### 3.2. GO and KEGG Pathway Analysis

**Figure 2:**
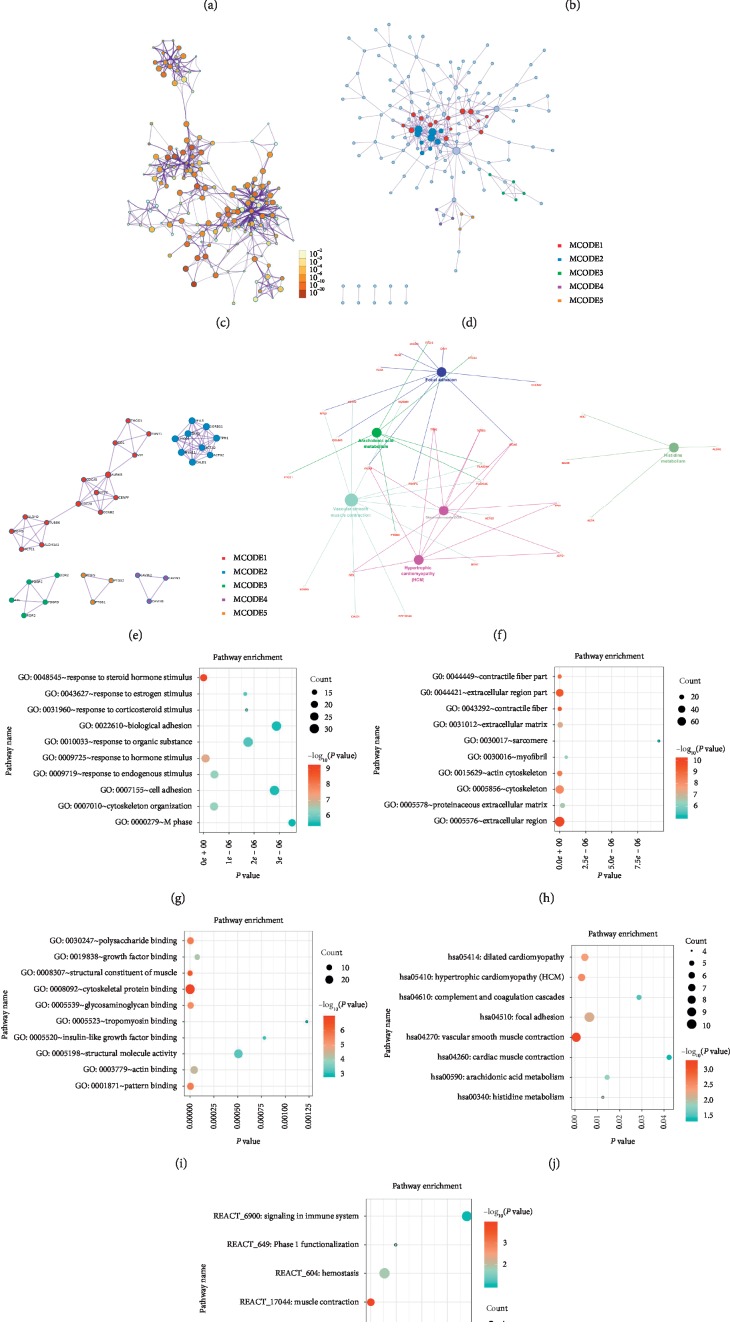
GO analysis and KEGG pathway analysis of DEGs. ((a), (b), (c), (d), (e)) GO analysis and KEGG pathway analysis from the Metascape website. (f) Illustration of the results of KEGG pathway analysis with the Clugo plugin in Cytoscape software. ((g), (h), (i) (j), (k)) Bubble diagram of BP, CC, MF, KEGG, and REACTOME analysis for BLCA. Significant pathways with *P* values < 0.01 were plotted by the R language.

To further analyse the potential functions of DEGs, GO analysis was performed on the DEGs by using Metascape online tools; we found that the DEGs were mostly enriched in the NABA core matrisome cellular component, muscle contraction, supramolecular fibre organization, muscle structure development, and tissue morphogenesis (Figures 2(a)–2(e)). Then, we performed GO analysis through the DAVID website and visualized the data with the R language. Concerning biological processes (BPs), the DEGs were enriched in response to steroid hormone stimuli, hormone stimuli, endogenous stimuli, and oestrogen stimuli and in cytoskeleton organization (Figure 2(g)). The changes in cellular components (CCs) were significantly enriched in the extracellular region, contractile fibre, extracellular region part, contractile fibre part, and actin cytoskeleton (Figure 2(h)). The changes in molecular function (MF) were significantly enriched in the cytoskeletal protein binding, structural constituent of muscle, pattern binding, polysaccharide binding, and glycosaminoglycan binding (Figure 2(i)). We further analysed the DEG-enriched REACTOME and KEGG pathways through the DAVID online tool and visualized it with the R language. We found that the DEG genes were mainly enriched in REACTOME pathways such as muscle contraction, haemostasis, phase 1 functionalization, biological oxidation, signalling by PDGF, and signalling in the immune system (Figure 2(k)). KEGG pathway analysis revealed that the hub gene was mainly enriched in vascular smooth muscle contraction, hypertrophic cardiomyopathy (HCM), dilated cardiomyopathy (DCM), focal adhesion, arachidonic acid metabolism, and histidine metabolism (Figure 2(j)). Then, we used the Clugo plugin for Cytoscape to illustrate the results of KEGG path analysis (Figure 2(f)).

### 3.3. Protein-Protein Interaction and Screening of Hub Genes

**Figure 3:**
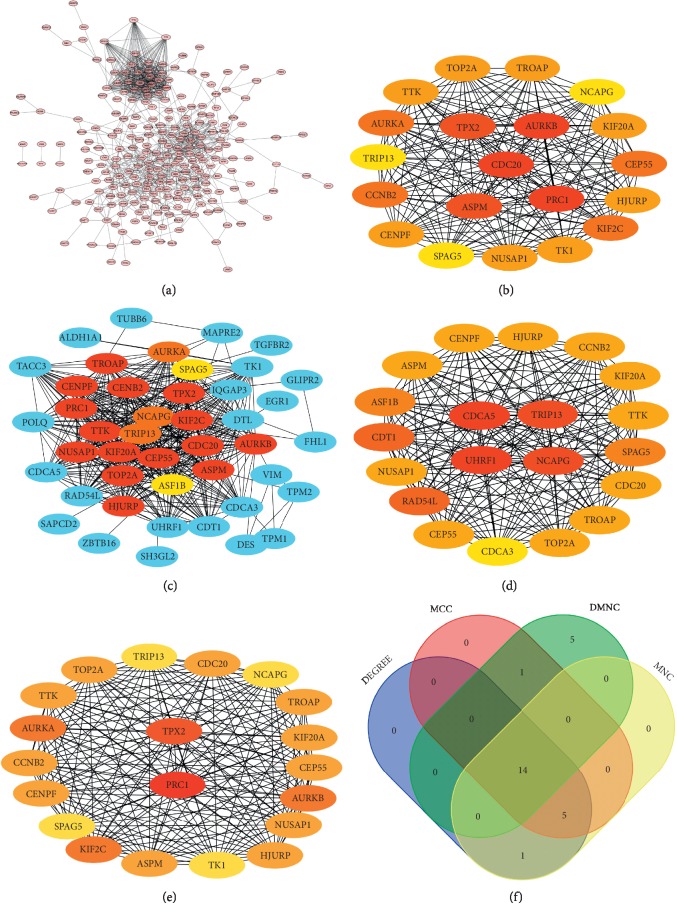
Determination of the hub genes. (a) PPI network of 291 promising target genes in BLCA. ((b), (c), (d), (e)) Four different metrics: DEGREE, MCC, DMNC, and MNC. (f) A Venn diagram was used to identify 14 hub genes in BLCA.

To better understand the relationship between DEGs, we used the STRING online tool to study the relationship between various DEGs (Figure 3(a)). Then, we identified 14 hub genes based on the DEGREE (Figure 3(b)), MCC (Figure 3(c)), DMNC (Figure 3(d)), and MNC (Figure 3(e)) plugins for the Cytoscape software. The 14 hub genes were ASPM, CCNB2, CDC20, CENPF, CEP55, HJURP, KIF20A, NCAPG, NUSAP1, SPAG5, TOP2A, TRIP13, TROAP, and TTK (Figure 3(f)).

### 3.4. Differential Expression Analysis of Hub Genes in BLCA and Normal Bladder Tissues

**Figure 4:**
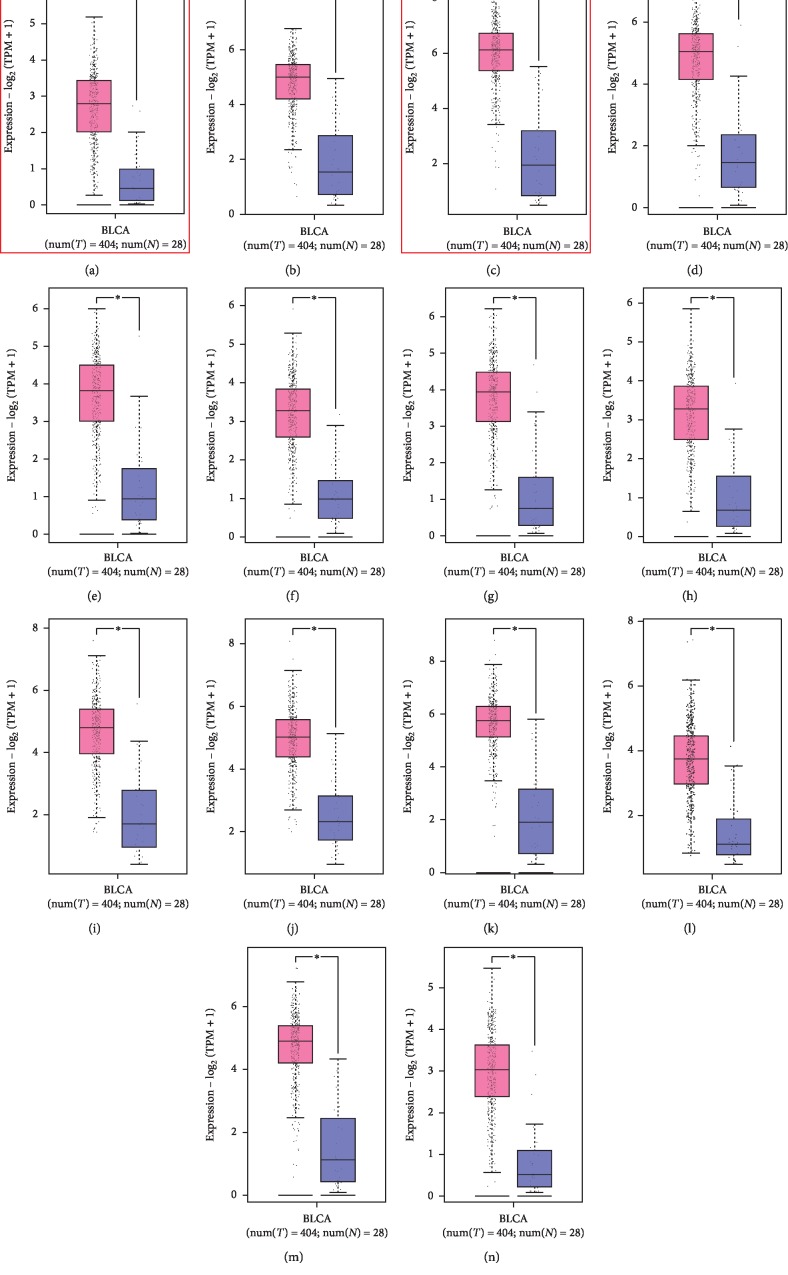
Expression analysis of 14 hub genes in BLCA based on GEPIA. (a) ASPM, (b) CCNB2, (c) CDC20, (d) CENPF, (e) CEP55, (f) HJURP, (g) KIF20 A, (h) NCAPG, (i) NUSAP1, (j) SPAG5, (k) TOP2A, (l) TRIP13, (m) TROAP, (n), and TTK; *P* < 0.05 was considered statistically significant.

To verify the differential expression of the hub genes between BLCA and normal bladder tissues, we analysed the 14 hub genes using the GEPIA website-based TCGA database. We found that ASPM (Figure 4(a)), CCNB2 (Figure 4(b)), CDC20 (Figure 4(c)), CENPF (Figure 4(d)), CEP55 (Figure 4(e)), HJURP (Figure 4(f)), KIF20A (Figure 4(g)), NCAPG (Figure 4(h)), NUSAP1 (Figure 4(i)), SPAG5 (Figure 4(j)), TOP2A (Figure 4(k)), TRIP1 3(Figure 4(l)), TROAP (Figure 4(m)), and TTK (Figure 4(n)) were significantly upregulated in BLCA tissue compared with normal bladder tissue, and the differences were statistically significant.

### 3.5. Determination of CDC20 and ASPM as the Target Genes by Survival Analysis

**Figure 5:**
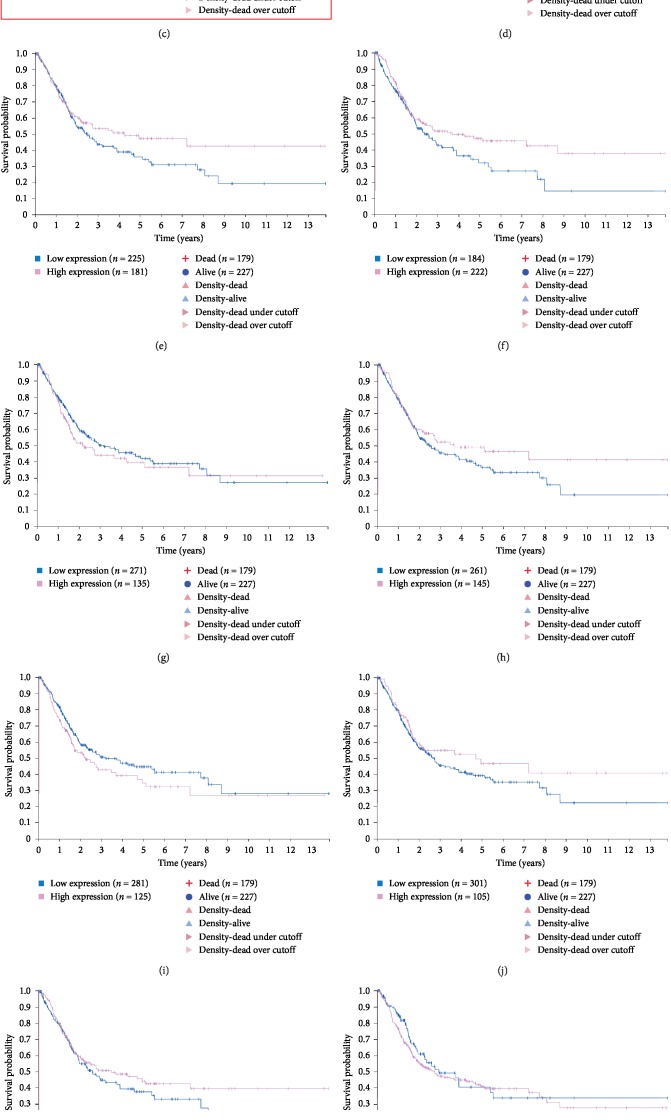
Survival analysis of the 14 hub genes in BLCA based on the Human Protein Atlas. (a) ASPM, (b) CCNB2, (c) CDC20, (d) CENPF, (e) CEP55, (f) HJURP, (g) KIF20A, (h) NCAPG, (i) NUSAP1, (j) SPAG5, (k) TOP2A, (l) TRIP13, (m) TROAP, (n), and TTK; *P* < 0.05 was considered statistically significant.

To investigate the relevance of the hub genes in BLCA patient survival, we performed a survival analysis of the hub genes using the Human Protein Atlas online tool for differential analysis (Figure 5(a)–5(n)). We found that the analysis of CDC20 and ASPM expression and survival was statistically significant in BLCA. The knot and the GEPIA websites give the expression of each hub gene; we chose CDC20 and ASPM as our target genes, and all of the genes with high expression status predicted poor prognosis.

### 3.6. The Biological Role of CDC20 in Tumours

**Figure 6:**
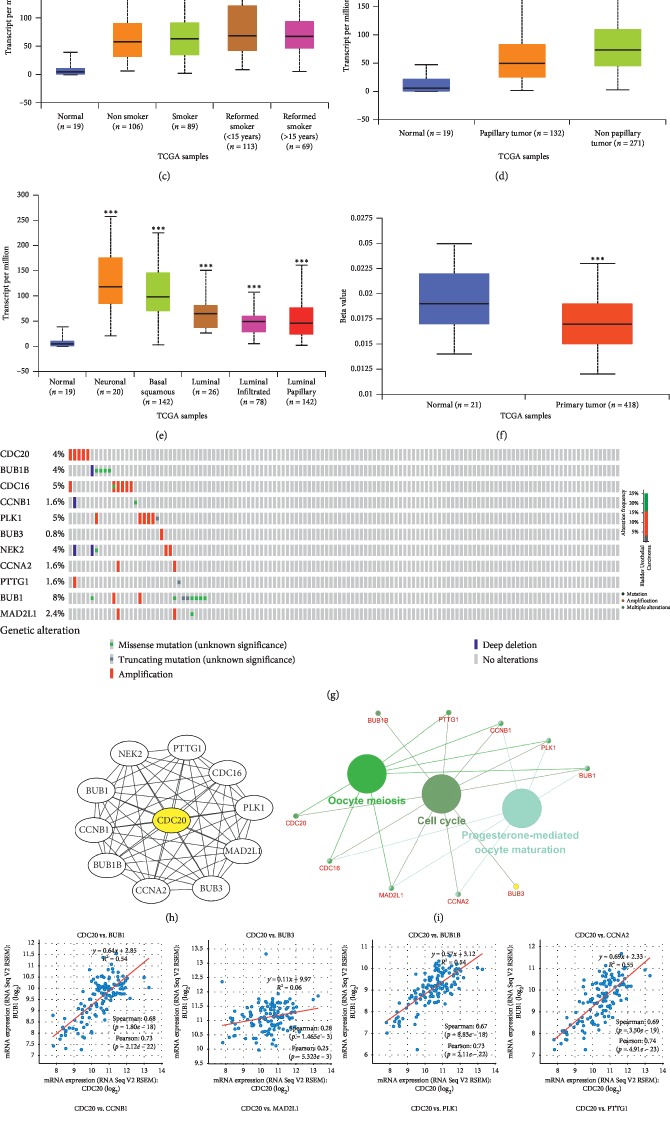
The biological role of CDC20 in tumours. (a) Expression of CDC20 in various tumours. (b) Expression of CDC20 in BLCA based on sample type. (c) Expression of CDC20 in BLCA based on patient smoking habits. (d) Expression of CDC20 in BLCA based on histological subtype. (e) Expression of CDC20 based on molecular subtypes of BLCA. (f) The promoter methylation level of CDC20 in BLCA.(g) Variation of CDC20-related genes in BLCA. (h) Interacting proteins for the CDC20 gene STRING interaction network preview (showing top 10 STRING interactants). (i) Illustration of the results of KEGG pathway analysis with the Clugo plugin in Cytoscape software. (j) A scatter plot showing the correlation between CDC20 expression and the 8 hub gene signature. ^*∗*^*P* < 0.05, ^*∗∗*^*P* < 0.01, ^*∗∗∗*^*P* < 0.001.

To investigate whether the CDC20 gene acts as an oncogene in other tumours, we analysed the differential expression of CDC20 in different tumours and normal tissues through the TIMER website. We found that CDC20 is upregulated in a variety of tumours, including BLCA, BRCA, CHOL, COAD, ESCA, HNSC, KICH, KIRC, KIRP, LIHC, LUAD, LUSC, PPAD, READ, STAD, THCA, and UCEC (Figure 6(a)). This suggests that the role of CDC20 in regulating the underlying mechanisms of tumorigenesis and progression is identical in different tumours. We found that CDC20 is highly expressed in BLCA through the UALCAN website (Figure 6(b)). We also found that CDC20 has differential expression in patients with different smoking habits (Figure 6(c)), histological subtypes (Figure 6(d)), and molecular subtypes (Figure 6(e)). Moreover, CDC20 is related to the promoter methylation level in BLCA (Figure 6(f)). To explore the underlying molecular mechanisms of CDC20, we first identified genes that have a protein-protein interaction with CDC20 via the STRING website (Figure 6(h)), and then through KEGG pathway analysis of these related genes, we found that CDC20-related genes are mainly enriched in the cell cycle, oocyte meiosis, and progesterone-mediated oocyte maturation (Figure 6(i)). We found that CDC20 has a strong coexpression relationship with BUB1, BUB3, BUB1B, CCNA2, CCNB1, MAD2L1, PLK1, and PTTG1 (Figure 6(j)).

### 3.7. The Biological Role of ASPM in Tumours

**Figure 7:**
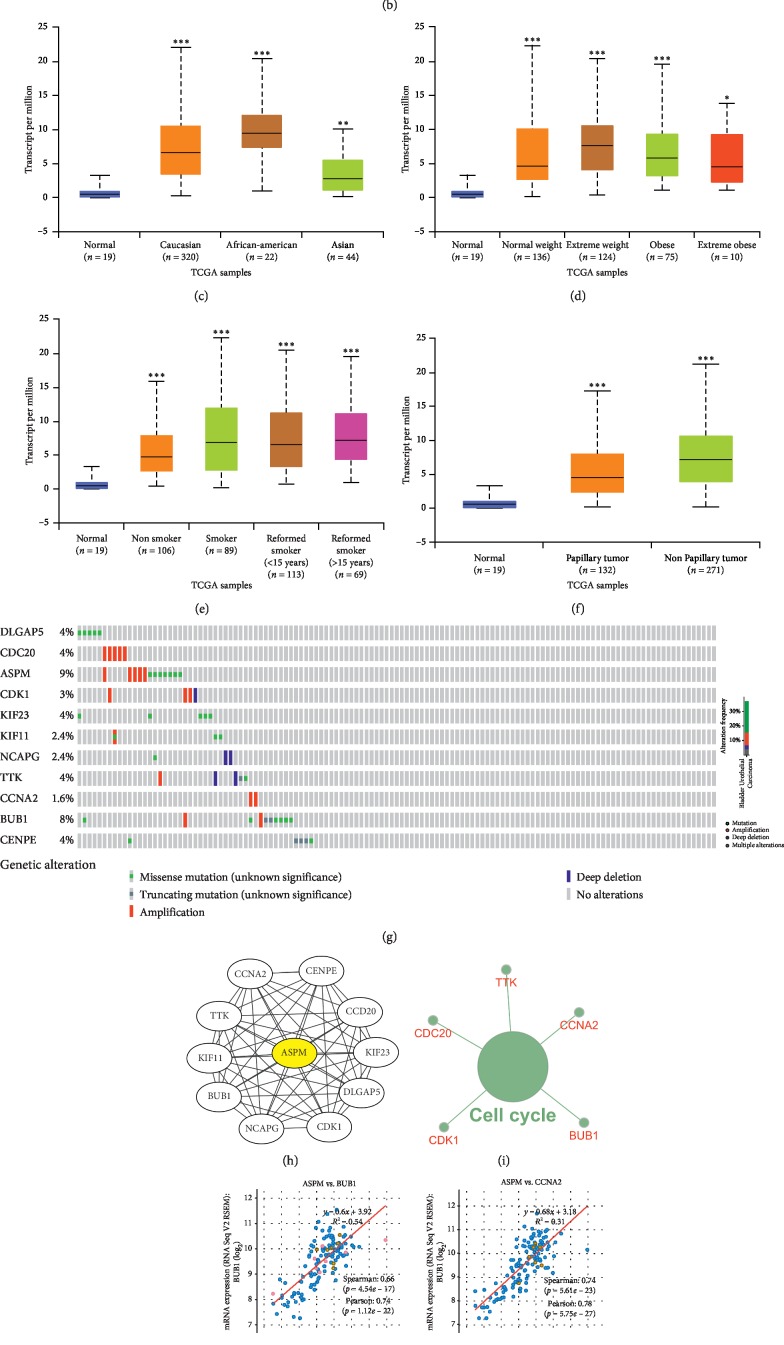
The biological role of ASPM in tumours. (a) Expression of ASPM in various tumours. (b) Expression of ASPM in BLCA based on sample type. (c) Expression of ASPM in BLCA based on the patient race. (d) Expression of ASPM in BLCA based on patient weight.(e) Expression of ASPM in BLCA based on patient smoking habits. (f) Expression of CDC20 in BLCA based on histological subtype.(g) Variation of ASPM-related genes in BLCA. (h) Interacting proteins for the ASPM gene STRING interaction network preview (showing top 10 STRING interactants). (i) Illustration of the results of KEGG pathway analysis with the Clugo plugin in Cytoscape software. (j) A scatter plot showing the correlation between ASPM expression and the 5 hub gene signature. ^*∗*^*P* < 0.05, ^*∗∗*^*P* < 0.01, ^*∗∗∗*^*P* < 0.001.

To investigate whether the ASPM gene acts as an oncogene in other tumours, we analysed the differential expression of ASPM in different tumours and normal tissues via the TIMER website. We found that ASPM is upregulated in a variety of tumours, including BLCA, BRCA, CHOL, COAD, ESCA, HNSC, KICH, KIRC, KIRP, LIHC, LUAD, LUSC, PPAD, READ, STAD, THCA, and UCEC (Figure 7(a)). This suggests that the role of ASPM in regulating the underlying mechanisms of tumorigenesis and progression is identical in different tumours. We found that ASPM is highly expressed in BLCA via the UALCAN website (Figure 7(b)). We also found that ASPM has differential expression in patients with different races (Figure 7(c)), weights (Figure 7(d)), smoking habits (Figure 7(e)), and histological subtypes (Figure 7(f)). To explore the underlying molecular mechanisms of ASPM, we first identified genes that have a protein-protein interaction with ASPM via the STRING website (Figure 7(h)). Then, through the KEGG pathway analysis of these related genes, we found that ASPM-related genes are mainly enriched in the cell cycle (Figure 7(i)). We found that ASPM has a strong coexpression relationship with BUB1, CCNA2, CDC20, CDK1, and TTK (Figure 7(j)).

### 3.8. CDC20 and ASPM Act as Immune-Related Genes in BLCA

**Figure 8:**
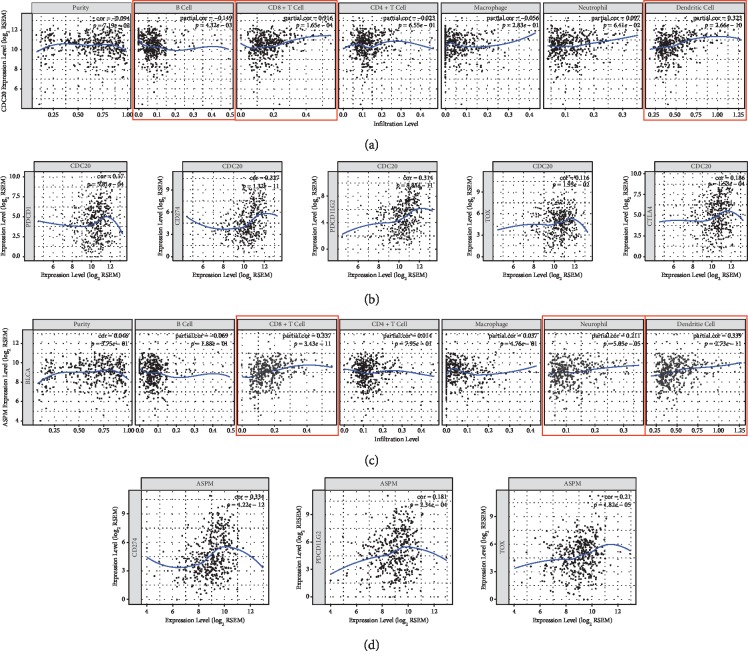
Tumour immune correlation analysis based on the TIMER website. (a) Relationship between CDC20 expression and immune cells. (b) Relationship between CDC20 expression and immune checkpoints. (c) Relationship between ASPM expression and immune cells. (d) Relationship between ASPM expression and immune checkpoints.

To investigate the relationship between CDC20, ASPM, and tumour immunity, we analysed the relationship between CDC20, ASPM, and immune cell infiltration via the TIMER website. We found that CDC20 is involved in the infiltration of B cells, CD8+ T cells, and dendritic cells in BLCA (Figure 8(a)). ASPM is involved in the infiltration of CD8+ T cells, neutrophils, and dendritic cells in BLCA (Figure 8(c)). Since immunotherapy is currently focused on immunological checkpoint inhibitors such as PDCD1, CD274, PDCD1LG2, TOX, and CTLA4, we further analysed the coexpression relationship of CDC20, ASPM, and immune checkpoint-related genes PDCD1, CD274, PDCD1LG2, TOX, and CTLA4. We were surprised to find that CDC20 has a significant coexpression relationship with PDCD1, CD274, PDCD1LG2, TOX, and CTLA4 (Figure 8(b)). ASPM has a significant coexpression relationship with CD274, PDCD1LG2, and TOX (Figure 8(d)). TOX is a newly discovered gene, and three consecutive articles published in Nature recently introduced the role of the TOX gene in tumour immunotherapy. Fortunately, our study found that CDC20, ASPM, and TOX also have strong coexpression relationships; the above points lay a very solid foundation for our future research.

### 3.9. In Vitro Cell Experiment

**Figure 9:**
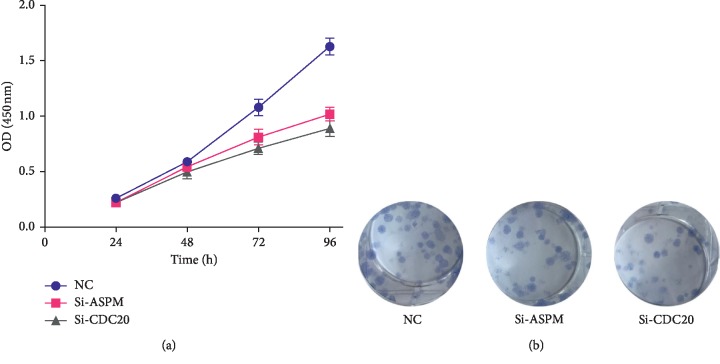
Experimental validation of ASPM and CDC20. (a) Cell Counting Kit-8 (CCK8) assay. (b) Clone formation assay. ^*∗∗∗*^*P* < 0.001. Student's *t*-tests were used to evaluate the statistical significance of differences.

In vitro, we interfered with the expression of ASPM and CDC20 and then used the cell counting kit-8 experiment and clone formation experiment to detect the effect on the proliferation of bladder cancer T24 cell line. The above two experimental results show that the proliferation rate of the siASPM and siCDC20 group is significantly lower than that of the siControl group (Figures 9(a) and 9(b)).

## 4. Discussion

Bladder cancer (BLCA) is a serious health problem worldwide and the second most common malignant tumour of all genitourinary tract tumours [1]. Unfortunately, until recently, the treatment of bladder cancer has progressed very little. For 30 years, clinicians have consistently used similar, limited treatments to serve patients. At present, transurethral resection of bladder tumours is the most common surgical procedure for noninvasive bladder cancer, but the recurrence rate is higher [23]. Therefore, there is a very urgent need to find new therapeutic strategies and biomarkers.

In this study, we found 291 integrated DEGs in BLCA by a comprehensive analysis of GEO (GSE7476, GSE13507) and TCGA BLCA datasets. The 291 integrated DEGs were then subjected to GO (BP, CC and MF) analysis. The DEG enrichment analysis produced the following terms: steroid hormone stimulus, hormone stimulus, cytoskeleton organization, endogenous stimulus, and oestrogen stimulus (BP); extracellular region, contractile fibre, extracellular region part, contractile fibre part, and actin cytoskeleton (CC); and cytoskeletal protein binding, structural constituent of muscle, pattern binding, polysaccharide binding, and glycosaminoglycan binding (MF). These results indicate that these DEGs are involved in the mitotic process and in the invasion and metastasis of bladder cancer cells. The REACTOME pathway analysis showed that DEGs are mainly enriched in the six pathways: muscle contraction, haemostasis, phase 1 functionalization, biological oxidation, signalling by PDGF, and signalling in the immune system. The KEGG pathway analysis showed that DEGs are mainly enriched in the following six pathways: vascular smooth muscle contraction, hypertrophic cardiomyopathy (HCM), dilated cardiomyopathy (DCM), focal adhesion, arachidonic acid metabolism, and histidine metabolism. Two different pathway enrichment algorithms showed that DEGs are involved in the process of muscle contraction, and biomechanics play a key role in the development of bladder cancer. Biomechanics is related to the deformability of cancer cells, which is involved in cell signalling, cell adhesion, migration, invasion, and metastatic potential. [24] Biomechanics is a discipline that uses mechanical theory to study the movement of matter in living organisms. Therefore, studying these pathways will help elucidate the underlying mechanisms of bladder cancer proliferation and invasion and help predict cancer progression. This is also related to immune system signalling, further confirming that decreased immune system function is closely related to tumorigenesis [25].

We constructed a PPI network with 291 integrated DEGs and identified the following 14 hub genes: ASPM, CCNB2, CDC20, CENPF, CEP55, HJURP, KIF20A, NCAPG, NUSAP1, SPAG5, TOP2A, TRIP13, TROAP, and TTK. Most of these factors affect the occurrence and development of cancer mainly by affecting the cell cycle. These hub genes can be used as therapeutic targets for bladder cancer. We then performed a prognostic analysis of these 14 hub genes using the GEPIA and Human Protein Atlas websites. Surprisingly, the expression levels of CDC20 and ASPM are associated with the prognosis of patients with bladder cancer. Therefore, we chose CDC20 and ASPM as target genes for this study.

CDC20 is associated with the meiotic cell cycle in oocytes, and APC is associated with the regulation of cell cycle. Human CDC20 is a homologue of the CDC20 protein and is generally considered to be one of the major regulators of mitosis. It is responsible for activating the APC/C complex, an E3 ubiquitin ligase that targets mitotic proteins such as securin and cyclin B for 26S proteasome degradation, allowing cells to exit mitosis [26]. Recent literature has reported that CDC20 is highly expressed in pancreatic cancer [27], colon cancer [28], osteosarcoma cancer [29], lung adenocarcinoma [30], oral squamous cell carcinoma, and hepatocellular carcinoma [31, 32]. Consistent with our research, increased expression of CDC20 in bladder cancer patients is associated with poor prognosis [33]. It has been reported that CDC20 may act as an oncoprotein to promote the progression and development of human cancer and that it is a promising therapeutic target [34]. We found that CDC20 is highly expressed in BLCA via the UALCAN website. We also found that CDC20 is differentially expressed in patients with different smoking habits, histological subtypes, and molecular subtypes. In addition, CDC20 is associated with promoter methylation levels in BLCA. To investigate the relationship between CDC20 and tumour immunity, we analysed the relationship between CDC20 and immune cell infiltration via the TIMER website, and we found that CDC20 is involved in the infiltration of B cells, CD8+ T cells, and dendritic cells in BLCA. We were surprised to find that CDC20 has a significant coexpression relationship with PDCD1, CD274, PDCD1LG2, TOX, and CTLA4.

ASPM is involved in the spindle tissue, spindle localization, and cytokinesis of all dividing cells, and the extreme C-terminus of the protein is required for ASPM localization and function. In addition, it may have a role in regulating neurogenesis [35]. The purpose of this study was to assess the critical role that ASPM plays in the development of cancer and determine whether it can be used as a biomarker for bladder cancer. Recent literature reports that ASPM expression disorders are associated with the progression of epithelial ovarian cancer [36], colorectal cancer [37], prostate cancer [38], and hepatocellular carcinoma [39]. Consistent with our research, increased expression of ASPM in bladder cancer patients is associated with poor prognosis [40]. We found that ASPM is highly expressed in BLCA via the UALCAN website. We also found that ASPM is differentially expressed in patients with different races, weights, smoking habits, and histological subtypes. To investigate the relationship between ASPM and tumour immunity, we analysed the relationship between ASPM and immune cell infiltration via the TIMER website, and we found that ASPM is involved in the infiltration of CD8+ T cells, neutrophils, and dendritic cells in BLCA. We were surprised to find that ASPM has a significant coexpression relationship with CD274, PDCD1LG2, and TOX.

Among these immune checkpoints, it is worth noting that a molecule called TOX is a newly discovered gene, and three consecutive articles published in Nature recently introduced the role of the TOX gene in tumour immunotherapy [41–43]. TOX is a key regulator of T cell dysfunction. TOX is specifically required for T cell differentiation in an environment of chronic antigen stimulation (e.g., tumours and chronic infections) [41]. Manipulation of TOX expression is thought to calibrate T cells to maintain their effector function in cell differentiation and ultimately achieve long-lasting therapeutic outcomes [42, 43]. We believe that CDC20 and ASPM may affect the development of bladder cancer by affecting TOX molecules.

In addition, we interfered with the expression of ASPM and CDC20 in vitro and then used the cell counting kit-8 experiment and clone formation experiment to detect the effect on the proliferation of bladder cancer T24 cell line. The above two experimental results show that the proliferation rate of the siASPM and siCDC20 group is significantly lower than that of the siControl group. In vitro experiments further support our conclusions.

## 5. Conclusions

In summary, our current study reveals two potential biomarkers for BLCA, CDC20, and ASPM. Both of these genes may be new immunotherapeutic targets for BLCA. In addition, the biological role of the ASPM and CDC20 molecules in the development of bladder cancer was confirmed by in vitro experiments. We continue to study the underlying mechanisms by using bioinformatics. The findings of this study may pave the way for the identification of other functions in vitro and in vivo by our group and others.

## Figures and Tables

**Table 1:** A total of 291 DEGs were identified from the TCGA and GEO datasets, including 50 upregulated and 241 downregulated genes in the comparison of BLAC tissues with normal tissues.

DEGs	Genes name
Upregulated genes	MTFP1, IQGAP3, ESM1, FASN, CDC20, HILPDA, PAFAH1B3, ETV4, TTK, PODXL2, NUSAP1, TPX2, CENPF, CDT1, AURKB, KIF20 A, SAPCD2, RAD54 L, KIF2C, HJURP, DTL, TROAP, TOP2A, NCAPG, ASPM, AURKA, PRC1, TK1, SYNE4, CDCA5, CA9, CDCA3, PFKFB4, SPAG5, TRIP13, ASF1B, CELSR3, UHRF1, TMEM74 B, CCNB2, POLQ, CEP55, IGSF9, TACC3, WDR72, ISG15, PRSS8, TNNT1, MMP1, TCN1

Downregulated genes	MOXD1, NDNF, ABCA8, SRPX, FGF9, FAM107 A, MFAP4, FOXF1, OLFM1, TCF21, CFD, SCARA5, PRAC1, PAMR1, FCER1A, CPED1, ADAMTS8, COL16A1, ASPA, SPON1, OLFML3, DCN, FGL2, COLEC12, TMEM119, PDGFC, DIXDC1, GLT8D2, DPT, RERGL, TCEAL2, MYH11, MRGPRF, SLC9A9, SDPR, GFRA1, BMP5, SMOC2, ALDH1A1, SPARCL1, ABI3BP, CNRIP1, EVA1C, PDGFD, ITM2A, CRISPLD2, GHR, CDH11, ADAMTS1, RERG, COX7A1, FLNC, HSPB6, PLAC9, TMOD1, OLFML1, HSD17B6, CYBRD1, SLIT2, JAM3, EMILIN1, CNN1, FHL1, ACTG2, LUM, BIN1, EGR2, PELI2, MAMDC2, CLIP3, ENPP2, ZEB2, RASL12, ITGA8, ACTA2, SORBS2, STON1, PDLIM3, CXCL12, LTBP4, C2orf40, NBEA, GPR183, GATA5, RNASE4, ANTXR2, SGCE, PLA2G4C, PAM, ZNF521, TSHZ3, PALLD, PGM5, ACOX2, NR2F1, EDNRA, PTGS1, ROR2, GYPC, TGFBR2, LHFP, PARM1, C1S, RGL1, ALDH2, ATP1A2, RGS1, WLS, TAGLN, CRYAB, KCNMB1, FZD7, PRUNE2, SERPINF1, SORBS1, MSRB3, SYNPO2, LMOD1, LPP, PTGIS, MAOB, DPYSL2, BOC, EMP3, SELM, PLSCR4, KLF9, DKK3, VIM, DES, RGS2, SYNM, PCP4, PRICKLE2, GAS6, JAZF1, PLA2G4A, CALD1, PDK4, HAND2-AS1, COL6A2, C7, ZCCHC24, ACTC1, GSTM5, AEBP1, TGFB3, FILIP1L, P2RX1, FXYD6, DDR2, RNF150, TIMP2, SH3GL2, WFDC1, BNC2, A2M, TPM2, CASQ2, DACT3, TCF4, TPM1, RARRES2, GLIPR2, DACT1, AXL, CAV1, MAPRE2, NDN, FERMT2, PRICKLE1, RBPMS2, PTRF, CPVL, CTGF, TNFAIP8L3, PTGDS, DOCK11, ACKR1, PCOLCE2, PRRT2, FAM162 B, HDC, CPE, EGR1, ZBTB16, EPDR1, SBSPON, DPYSL3, MYL9, CPXM2, COL6A3, CSRP1, HOXA13, MYOM1, FOS, MGP, DUSP1, ANGPTL2, AQP1, IGFBP6, NFIA, MFAP5, CCL19, EPB41L3, SFRP2, MAP1B, GAS1, CAP2, CCL2, FLNA, DKK1, C8orf4, C3, NFIB, CLIC4, AGR3, RASD1, ZFP36, NEXN, TGFB1I1, FOSB, FBLN2, PPP1R14 A, PRKCDBP, TUBB6, REEP1, C11orf96, FAM129 A, SMTN, APOD, CYR61, SERPINA3, HSPB8, CKB, IGFBP2, SFRP1, ITGA5, PTGS2, NUPR1, AHNAK2

## Data Availability

The data used to support the findings of this study are available from the corresponding author upon request.
